# Lo spettro dei test di funzionalità tiroidea durante il ricovero per infezione da SARS-COV-2

**DOI:** 10.1007/s40619-022-01020-9

**Published:** 2022-02-23

**Authors:** Lisa Buci, Alessandro Peri

**Affiliations:** 1 Patologie ipotalamo-ipofisarie e alterazioni del sodio unit, A.O.U. Careggi, Firenze, Italia; 2 Dipartimento di Scienze Biomediche, Sperimentali e Cliniche Mario Serio, Università di Firenze, Firenze, Italia

Commento a:

**The spectrum of thyroid function tests during hospitalization for SARS COV-2 infection.**

**I. Campi, I. Bulgarelli, A. Dubini, G.B. Perego, E. Tortorici, C. Torlasco, E. Torresani, L. Rocco, L. Persani, L. Fugazzola.**

**Eur J Endocrinol (2021) 184(5):699–709**

La pandemia che stiamo vivendo ci sta portando a scoprire i numerosi effetti che il SARS-Cov-2 può avere sull’organismo umano e, tra di essi, molta enfasi è stata data alle disfunzioni tiroidee. In particolare, si è evidenziato come la ghiandola tiroidea possa essere facilmente bersaglio dei coronavirus perché esprime l’enzima di conversione dell’angiotensina 2 (e, infatti, il suo coinvolgimento era già stato dimostrato all’istologia durante l’epidemia di SARS-Cov-1 e confermato col SARS-Cov-2) [1], con possibili conseguenti processi distruttivi tipici di una tiroidite subacuta. Sulla base di questa premessa, molti studi hanno ricondotto il riscontro di bassi valori di TSH in pazienti ospedalizzati per infezione da SARS-Cov-2 a una tireotossicosi da distruzione ghiandolare, nonostante la frequente assenza della tipica sintomatologia dolorosa al collo e dell’elevazione delle frazioni libere ormonali, e il rapido ripristino di eutiroidismo biochimico non coerente col tipico corso trifasico (tireotossicosi, ipotiroidismo ed eutiroidismo) della tiroidite subacuta. In questi studi non è stata dosata la tireoglobulina (Tg), marker affidabile di tiroidite distruttiva indipendentemente dalla sua patogenesi.

D’altra parte, al momento della stesura del lavoro preso in esame, la letteratura riportava almeno 10 casi di tireotossicosi con tutti i tipici caratteri (sintomatologici, ecografici e laboratoristici) della tiroidite di De Quervain subacuta, probabilmente collegata all’infezione da COVID-19, con esordio da 2 a 5 settimane dopo la risoluzione della malattia virale [2].

È inoltre noto che nella sepsi e nei pazienti con sindrome da distress respiratorio si osservano modifiche dell’assetto laboratoristico tiroideo riferibili a una *non-thyroidal illness* (NTI) come conseguenza di un processo infiammatorio acuto, che agisce sia sulle attività delle deiodinasi (effetto periferico, per lo più attivando la 3 e inibendo la 2) che sull’asse ipotalamo-ipofisi-tiroide (effetto centrale, con effetto inibitorio). Nella NTI si apprezza una riduzione dell’ft3 con ft4 normale/lievemente ridotto e TSH sierico solitamente normale, ma che tende a ridursi nelle condizioni croniche più gravi.

Il lavoro preso in esame cerca di trovare la spiegazione più logica alle alterazioni dei test di funzionalità tiroidea osservati durante l’infezione da SARS-Cov-2.

Sono stati arruolati 144 pazienti consecutivi ricoverati per polmonite da COVID-19 in terapia intensiva o subintensiva e, tra essi, sono stati esclusi coloro che presentavano una pregressa diagnosi di distiroidismo e/o che avevano ricevuto un carico di iodio. La Tg e gli anticorpi anti-Tg (TgAb) sono stati misurati retrospettivamente sui campioni di siero dei pazienti con TSH ridotto/soppresso, così come la cortisolemia, la proteina C reattiva (PCR) e l’IL-6.

Il primo dato rilevante è che il 48% (55/115) dei pazienti non ha avuto alcuna alterazione dei valori di TSH, ft4 e ft3 sia al momento del ricovero che durante il follow-up.

Il 18% (21/115) ha presentato alterazioni delle frazioni libere ormonali in presenza di livelli normali di TSH. Il 10,4% (12/115) al momento del ricovero e il 23,5% (27/115) durante il ricovero ha presentato livelli di TSH inferiori alla norma, per lo più associati a normali livelli di fT4 e bassi livelli di fT3.

I livelli di Tg sono risultati costantemente normali con TgAb negativi nei pazienti con basso TSH, sia al momento del ricovero che durante il follow-up.

Alla dimissione per guarigione il quadro laboratoristico appariva normalizzato, al contrario di quanto accadeva nei pazienti deceduti. I bassi valori di TSH e ft3 correlavano inversamente con i livelli di PCR, cortisolemia e IL-6.

Nello studio sono stati inclusi due pazienti tiroidectomizzati; anch’essi hanno presentato riduzione del TSH.

L’infezione da SARS-Cov-2 è caratterizzata da una tempesta di citochine e vari studi riportano una diminuzione dei livelli di TSH, fT3 e del rapporto fT3/fT4, parallelamente alla gravità dell’infezione da COVID19, con livelli più bassi nei pazienti deceduti. La normalità della Tg in tutti i pazienti di questo studio e il quadro laboratoristico nei due pazienti tiroidectomizzati (evidentemente non riferibile a tireotossicosi da distruzione ghiandolare), nonché la correlazione inversa con PCR, IL6 e cortisolemia, sembrano ricondurre ragionevolmente il quadro laboratoristico più alla tempesta citochinica con effetti centrali e periferici dell’ambiente infiammatorio che non a un processo destruente.

Gli autori riconoscono che il principale punto debole di questo studio è l’assenza di un gruppo di controllo. Inoltre, da un punto di vista pratico, suggeriscono che: 1) poiché le alterazioni dei test di funzionalità tiroidea sono transitorie, è sconsigliabile la valutazione della funzione tiroidea durante il ricovero in terapia intensiva o subintensiva di pazienti affetti da COVID-19, poiché i risultati potrebbero essere fonte di diagnosi errate e spreco di risorse; e 2) il monitoraggio clinico e biochimico dopo la dimissione è appropriato, poiché è dimostrato che una tiroidite subacuta può complicare la fase di recupero di questi pazienti.
